# The Application of Enhanced Recovery After Surgery Protocols in Patients With Perforated Duodenal Ulcer

**DOI:** 10.7759/cureus.35760

**Published:** 2023-03-04

**Authors:** Satyajeet G Pathrikar, Gauri S Jadhav, Geet R Adhikari

**Affiliations:** 1 Department of Surgery, Anantshree Multispeciality Hospital, Aurangabad, IND; 2 Department of Surgery, Om Multispeciality Hospital, Malkapur, IND

**Keywords:** surgery, hollow viscous perforation, perforated peptic ulcer, perforated duodenal ulcer, enhanced recovery after surgery, eras protocols, emergency and elective surgery

## Abstract

Background

Enhanced recovery after surgery (ERAS) protocols are nowadays considered the standard of care for various elective surgical procedures. However, its utilization remains low in tier-two and tier-three cities of India, and there exists a significant variation in the practice. In the present study, we have investigated the safety and feasibility of these protocols or pathways in emergency surgery for perforated duodenal ulcer disease.

Methods

A total of 41 patients with perforated duodenal ulcers were randomly divided into two groups. All the patients across the study were treated surgically with the open Graham patch repair technique. Patients in group A were managed with ERAS protocols, while patients in group B were managed with conventional peri-operative practices. A comparison was established between the two groups in terms of the duration of hospital stay and other postoperative parameters.

Results

The study was conducted on 41 patients who presented during the study period. Group A patients (n=19) were managed with standard protocols, and group B patients (n=22) were managed with conventional standard protocols. As compared to the standard care group, patients in the ERAS group showed quicker postoperative recovery and lesser complications. The need for nasogastric (NG) tube reinsertion, postoperative pain, postoperative ileus, and surgical site infections (SSI) were all significantly lower in the patients of the ERAS group. A significant reduction in the length of hospital stay (LOHS) was found in the ERAS group when compared to the standard care group (relative risk {RR}=61.2; p=0.000).

Conclusions

The application of ERAS protocols with certain modifications in the management of perforated duodenal ulcers yields significant outcomes in terms of reduced duration of hospital stay and fewer postoperative complications in a selected subgroup of patients. However, the application of ERAS pathways in an emergency setup needs to be further evaluated to develop standardized protocols for a surgical emergency group of patients.

## Introduction

Enhanced recovery after surgery (ERAS) protocols, also known as fast-track protocols, were initially introduced by Dr. Henrik Kehlet. These protocols are based on an evidence-based modification of peri-operative care elements, which are focused on reducing physiological and psychological stress resulting in the enhanced recovery of the patients [1]. ERAS protocols are now a standard of care in many surgical specialties for various surgical procedures as they have demonstrated reduced length of hospital stay (LOHS) and postoperative mortality and morbidity [2-4].

Recently conducted studies have demonstrated the successful application of ERAS protocols in elective upper and lower gastrointestinal surgery. These studies showed beneficial effects such as the decreased incidence of postoperative nausea and vomiting (PONV), decreased postoperative ileus, early initiation of postoperative feeding, and reduction in total length of hospital stay [5,6].

Although considered to be safe and effective in elective procedures, the application and impact of ERAS protocols on emergency abdominal surgeries are still under study all over the world [7]. Conventional postoperative care fundamentals such as delayed removal of a nasogastric (NG) tube and late resumption of oral feeds due to fear of a leak at the repair site are still being practiced in major parts of India resulting in longer hospital stays. A recent randomized controlled trial (RCT) conducted on 47 patients diagnosed with a perforated duodenal ulcer who underwent laparoscopic Graham patch repair demonstrated a reduction of up to two days in the duration of hospital stay with no significant difference in the rates of complication with the implementation of ERAS pathways [8].

This study aimed to evaluate the efficacy, safety, and feasibility of ERAS protocols in patients undergoing emergency abdominal surgery for perforated duodenal ulcers. The present study was conducted in a tier-two city in India. Perforated duodenal ulcer is a common surgical emergency in the region, and the management is carried out by standardized procedure by either open or laparoscopic technique. The purpose of the study was to confirm the already established protocols in the management of patients undergoing open surgery for perforated duodenal ulcers, which will add to the already existing literature.

## Materials and methods

Methodology

The current study is a prospective randomized controlled study that assesses the impact of ERAS protocols in patients diagnosed with perforated duodenal ulcers undergoing emergency open Graham patch repair surgery. The study was approved by the Anantshree Hospital and Research Center Ethics Committee for one year from June 2021 onward (approval number: ECR/513/INST/MH2021-RR58). Written informed consent was obtained from all the participants with the freedom to withdraw at any time from the study. The data was collected on the standard form prepared by the researchers. The sample size was calculated by the statistician. The operating surgeon was blinded at the time of the operation. A resident surgeon who was not part of the study was assigned to collect data to avoid any kind of bias. The data collected was entered in proforma, tabulated, and analyzed with the Statistical Package for Social Sciences (SPSS) version 24.0 (IBM SPSS Statistics, Armonk, NY).

Patient enrolment

All patients clinically diagnosed with perforated duodenal ulcer and supported by relevant investigations admitted in the emergency department of Anantshree Hospital, Aurangabad, India, were included in the study after providing informed valid consent. The exclusion criteria of the study were as follows: age of <18 years, pregnant patients, spontaneously sealed off perforation not requiring any surgical intervention, those who had multiple perforated ulcers, large perforation with a size of >1 cm, patients undergoing any additional surgical procedures, patients with multiple comorbidities (American Society of Anesthesiologists {ASA} categories 3 and 4), and patient not willing to participate in the study.

Randomization

Patients were operated on in an emergency setting within 24 hours of admission and were randomly divided into two groups to be managed under ERAS protocols (group A) or standard care pathway (group B) in 1:1 ratio. As per their hospital registration number, patients were randomized into group A and group B using a simple random sampling technique via the Statistical Package for Social Sciences (SPSS) version 24.0 (IBM SPSS Statistics, Armonk, NY). The operating team was not informed about the sampling (the surgeon was blinded).

Preoperative care

Detailed history taking, clinical examination, and preoperative investigations were uniformly done across the study groups. All the patients were subjected to preoperative preparation, which included the placement of nasogastric (NG) tubes and foley catheters and initial resuscitation with intravenous (IV) fluids. Non-opioid multimodal analgesia (IV acetaminophen and lumbar epidural analgesia) was used in ERAS group patients.

Intraoperative care

All patients were resuscitated, and an exploratory laparotomy was carried out with the repair of duodenal perforation with an open Graham patch under general anesthesia. Group A patients received short-acting opioids only when necessary. Epidural catheters were used in appropriate patients. Multimodal non-opioid analgesia was used. Group B patients received intraoperative care under standard anesthetic protocols. After thorough peritoneal lavage, an abdominal drain was placed in the subhepatic space before closure.

Postoperative care

The patients belonging to Group A received postoperative care based on principles of the ERAS protocol, which included the use of non-opioid multimodal analgesia, use of opioids only for breakthrough pain, early initiation of nutrition, and early ambulation of the patients. Non-opioid multimodal analgesia was used for pain control. Patients inserted with an epidural catheter were ambulated after the removal of the epidural catheter at 24 hours postoperatively. The NG tube was removed when the drainage was less than 300 ml/day irrespective of the postoperative day or the absence of bowel sounds. The Foley catheter was removed when urine output was adequate over 24 hours. Drains were removed when the drainage was less than 100 ml/day irrespective of the resumption of oral feeds. The patients were allowed oral sips on day 1 after the first appearance of bowel sounds with a gradual shift to an unrestricted volume of liquids after 12 hours; semisolid food was started after 24 hours. Patients were shifted to oral analgesics on postoperative day 2 (POD2). Ambulation was started on postoperative day 1. They continued to receive IV antibiotics and proton pump inhibitors (PPIs) during their hospital stay.

Group B patients received postoperative care based on standard protocols. The Foley catheter was removed when the urine output was adequate over 24 hours. The NG tube was removed when the drainage was less than 100 ml/day with signs of resolution of the ileus after surgery. Drains were removed when a solid diet was tolerated for 24 hours. The patients were kept nil per oral (NPO) until the resolution of the ileus (passage of flatus) and started on a restricted volume of oral sips of clear fluids. They were gradually shifted to a soft diet followed by a normal diet as tolerated. Patients were given maintenance intravenous fluid, which was adjusted according to the fluid losses. They continued to receive IV antibiotics and proton pump inhibitors (PPIs) during their hospital stay. IV antiemetics and analgesia were given, and later, they were shifted to oral analgesia when they were started on a liquid diet.

In both groups, patients were admitted in ward or ICU based on the clinical scenario. Oral intake was stopped immediately in both groups whenever patients showed features of intolerance to the diet. The feeding was reinitiated after the symptoms had completely subsided and active bowel sounds reappeared. All patients were discharged in vitally stable condition when they could tolerate a solid diet and passed stool provided they showed no complications such as fever, wound infection, or leak. All patients were discharged with oral analgesia and were advised to continue oral PPIs (40 mg twice a day {BD}) for three months. They were called for follow-up at regular intervals over three months.

Endpoints

The primary endpoint of the study is the length of hospital stay. The average length of hospital stay was five to seven days, and a stay of more than a week was considered as prolonged hospital stay. The secondary endpoints included pain score on the visual analog scale (VAS), postoperative nausea and vomiting (PONV), duration of postoperative ileus (in days), surgical site infections (SSI), and need for nasogastric tube reinsertion.

## Results

**Figure 1:**
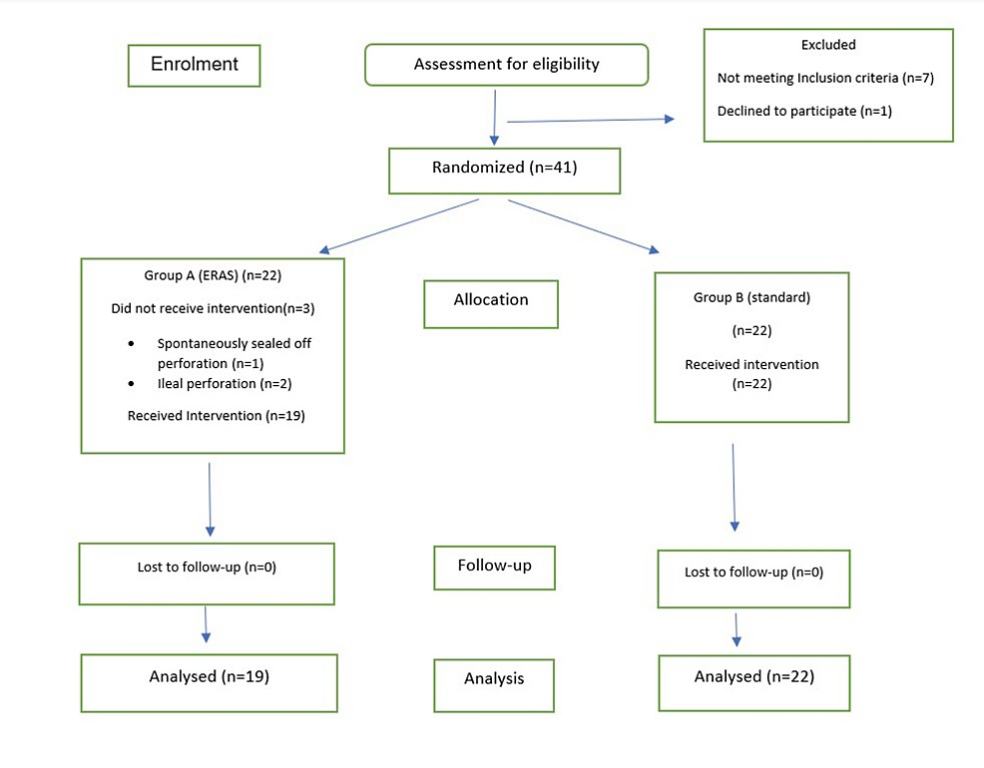
Enrolment process details as a Consolidated Standards of Reporting Trials (CONSORT) flow diagram ERAS: enhanced recovery after surgery

The study was conducted on 41 patients who presented during the study period, were diagnosed with duodenal perforation, and underwent open Graham patch repair surgery for the same. The details of the enrolment process are shown in the Consolidated Standards of Reporting Trials (CONSORT) flow diagram (Figure 1).

The patients were allotted to the standard protocol group B (n=22) and the ERAS protocol group A (n=19). The mean age of the patients was 48.17±14.73 years, the minimum age was 24 years, and the maximum age was 78 years. The majority of cases (26 {63.4%}) belonged to the more than 40 years age group, and 15 (36.6%) belonged to the less than 40 years age group; 38 (92.7%) were male, and three (7.3%) were female. As shown in Table 1, no significant association was found between study groups with respect to the age group and gender of the patients.

**Table 1:** Age and gender distribution of study participants in the study group ERAS: enhanced recovery after surgery

	Age group		Gender	
	≤40 years	>40 years	Male	Female
Group A (ERAS)	46.7%	46.2%	47.4%	33.3%
Group B (standard)	53.3%	53.8%	52.6%	66.7%
	p	0.975	p	0.639

**Figure 2:**
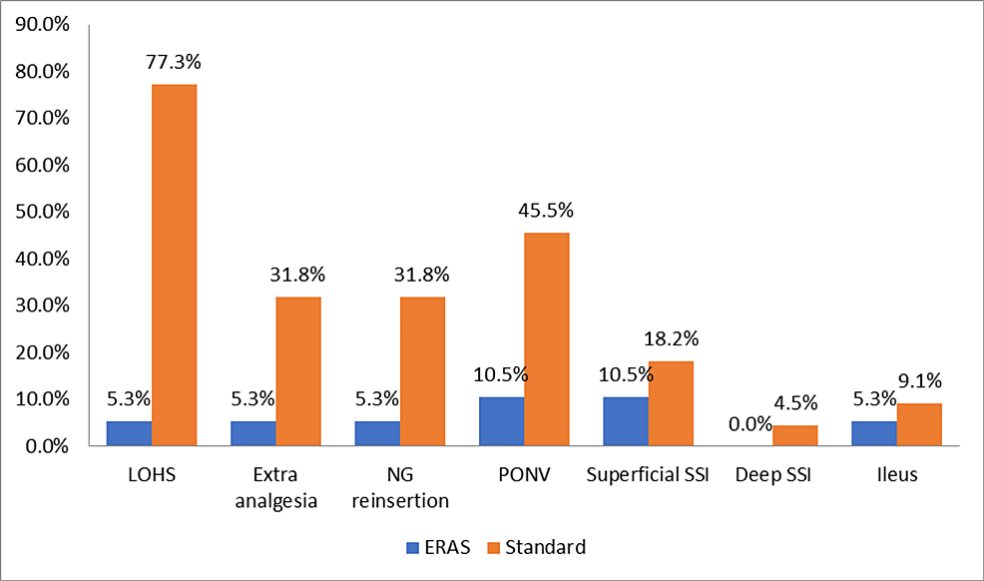
Outcome variables of study participants according to groups LOHS, length of hospital stay; ERAS, enhanced recovery after surgery; NG, nasogastric; PONV, postoperative nausea and vomiting; SSI, surgical site infection

As seen in Table 2, a significant reduction in the length of hospital stay was found in the ERAS group A when compared to the standard care group B (relative risk {RR}=61.2; p=0.000). The need for extra analgesia was significantly reduced in group A when compared to group B (RR=8.40; p=0.03). The need for nasogastric tube reinsertion was significantly reduced in group A when compared to group B (RR=8.40; p=0.032). Postoperative nausea and vomiting (PONV) were significantly lower in group A as compared to group B (RR=6.008; p=0.014). The incidence of postoperative ileus was seen in 5.3% in group A and 9.1% in group B. A total of 10.5% of patients developed superficial surgical site infection (SSI) in group A, and 18.2% developed it in group B, which was managed with dressing and local wound care. Only one person developed deep SSI in the study who belonged to the standard care group. The patient had a burst abdomen without any leak, and the patient was managed accordingly (Figure 2).

**Table 2:** Outcome variables of study participants according to the study groups ERAS, enhanced recovery after surgery; NG, nasogastric; PONV, postoperative nausea and vomiting; SSI, surgical site infection; RR, relative risk

		Group				RR	P value
		Group A (ERAS)		Group B (standard)			
Length of hospital stay	≤5 days	18	94.7%	5	22.7%	61.2	0.000
	>5 days	1	5.3%	17	77.3%		
Extra analgesia	Yes	1	5.3%	7	31.8%	8.40	0.032
	No	18	94.7%	15	68.2%		
NG reinsertion	Yes	1	5.3%	7	31.8%	8.40	0.032
	No	18	94.7%	15	68.2%		
PONV	Yes	2	10.5%	10	45.5%	6.008	0.014
	No	17	89.5%	12	54.5%		
Superficial SSI	Yes	2	10.5%	4	18.2%	0.478	0.489
	No	17	89.5%	18	81.8%		
Deep SSI	Yes	0	0.0%	1	4.5%	0.525	0.347
	No	19	100.0%	21	95.5%		
Ileus	Yes	1	5.3%	2	9.1%	1.80	0.639
	No	18	94.7%	20	90.9%		

The mean visual analog scale (VAS) score of 6.95±1.32 for postoperative pain was significantly higher (p=0.000) in group B as compared to group A, which was 3.74±1.48. The mean length of hospital stay of 7.82±3.59 days was significantly higher (p=0.000) in group B as compared to group B, which was 4.26±0.73 days. The mean duration (in days) of return to bowel activity of 2.64±0.90 days was significantly higher (p=0.000) in group B as compared to group A, which was 1.58±0.69 days (Table 3).

**Table 3:** Comparison of outcome variables (endpoints of the study) VAS, visual analog scale; ERAS, enhanced recovery after surgery; n, number of patients

Outcome	Group	n	Mean	P value
Postoperative pain score on VAS	A (ERAS)	19	3.74±1.48	0.000
	B (standard)	22	6.95±1.32	
Length of hospital stay (days)	A (ERAS)	19	4.26±0.73	0.000
	B (standard)	22	7.82±3.59	
Return of bowel activity (days)	A (ERAS)	19	1.58±0.69	0.000
	B (standard)	22	2.64±0.90	

## Discussion

The length of hospital stays and postoperative complications are considered important determinants to assess the impact of ERAS protocol after any major surgery. Early postoperative recovery is concordant with a successful implementation of the ERAS protocol. The patient’s overall mental, emotional, and physical well-being aids in long-term recovery. Various previous studies have shown the successful implementation of ERAS protocols in elective upper gastrointestinal and colorectal surgeries [5,6]. Recently, few studies have been conducted based on the application of ERAS protocols in an emergency setting. The possible effectiveness, safety, and feasibility of ERAS protocols were demonstrated successfully in randomized controlled studies conducted by Lohsiriwat [9] in colorectal emergency surgery and by Gonenc et al. [8], Mohsina et al. [10], and Masood [11] in upper gastrointestinal emergency surgery. As the aforementioned studies were based on elective procedures, the presence of peritonitis may have been underestimated. Many parameters of ERAS protocols for elective surgery are subject to application in case of emergency surgery [12].

**Figure 3:**
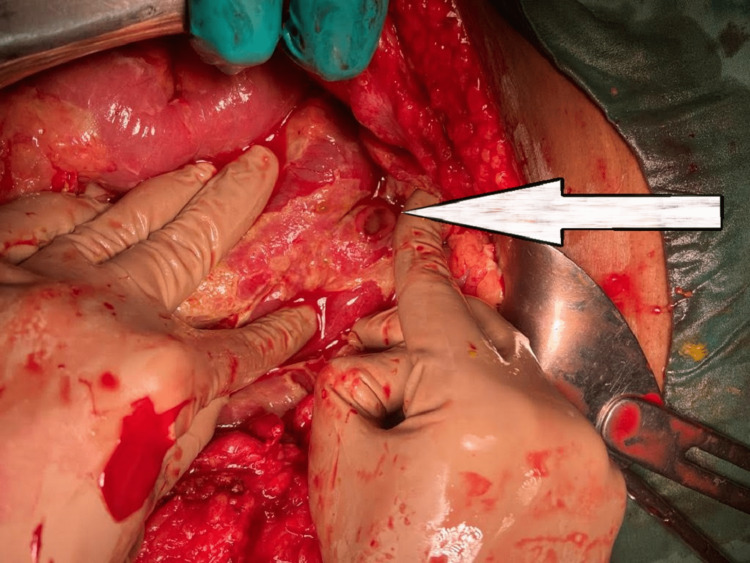
Intraoperative clinical picture showing perforated duodenal ulcer

Graham patch repair [13] was done in all patients in the present study. Patients with a perforation size of >1 cm were excluded from the study as they may require an adjuvant procedure. Furthermore, such patients are prone to the development of postoperative morbidity and mortality. This is similar to the exclusion criteria used in a study done by Gonenc et al. [8]. Figure 3 demonstrates intraoperative duodenal perforation.

Gastrointestinal motility may take a few days to recover because of diffuse peritonitis; hence, patients undergoing repair of perforated peptic ulcer are subjected to nasogastric decompression for at least 48 hours and are kept nil by mouth for three days in the postoperative period [14].

A meta-analysis study by Nelson et al. [15] reported that the use of nasogastric decompression does not always prevent aspiration and may even cause an increased incidence of postoperative complications. Studies have shown the safety of the omission of nasogastric decompression in an elective setup [1-6]. The safety of such omission for an emergency setup is still being studied. In their study, Gonenc et al. [8] removed the nasogastric tube immediately after the patient’s recovery from anesthesia, while Lohsiriwat [9] supported the idea of early removal if the drainage was less than 400 ml for 24 hours in patients undergoing emergency colectomy. In the present study, the NG tube was withdrawn if drainage was ≤300 ml over a period of 24 hours. Compared to the standard care of the management, the patients with the early removal of nasogastric tubes can be shifted early on oral liquids and show reduced incidence of postoperative ileus [6-10]. Gonenc et al. reported a mean duration of 1.5 days to start on oral clear fluids after the repair of the perforated peptic ulcer [8]. In the present study, the incidence of postoperative ileus was seen in 5.3% in the ERAS group and 9.1% in the standard care group. The mean duration (in days) of return to bowel activity of 2.64±0.90 days was significantly higher (p=0.000) in the standard method as compared to ERAS, which was 1.58±0.69 days.

The use of regional blocks, the use of general±epidural anesthesia, and avoiding long-acting pre-anesthetic medications play a key role in ERAS. The adequate control of pain with multimodal analgesia and avoiding opioid analgesia is the cornerstone in the successful implementation of ERAS protocols. This helps in decreasing the incidence of postoperative nausea and vomiting [1-6]. In their study, Gonenc et al. relied upon the use of nonsteroidal anti-inflammatory drugs (NSAIDs) for the management of postoperative pain with opioids for breakthrough pain [8]. The use of opioid analgesia was significantly reduced in our study, and the results are comparable to previous studies [8,9]. The mean VAS score of 6.95±1.32 for postoperative pain was significantly higher (p=0.000) in the standard method as compared to ERAS, which was 3.74±1.48. The need for extra analgesia was significantly reduced in the ERAS group when compared to the standard care group (RR=8.40; p=0.03). Postoperative nausea and vomiting (PONV) were significantly lower in the ERAS group as compared to the standard care group (RR=6.008; p=0.014). The results are comparable to the previous study, and thus, the use of opioid analgesia and routine nasogastric decompression can increase the duration of ileus and delay the initiation of oral feeding after major abdominal surgery [8-11,14,15].

Wisely and Barclay reported a significant reduction in postoperative complications in patients undergoing emergency laparotomy during the post-ERAS period [12]. Lohsiriwat concluded no significant reduction in the overall complication rates in patients [9]. Gonenc et al. concluded no significant difference in postoperative complication, readmission, or reoperation rates [8]. In the present study, there was a significant reduction in the rates of superficial SSI and PONV in the ERAS group.

The successful application of ERAS protocols has resulted in a significant decrease in the length of hospital stay (LOHS) in elective setups [1-7]. A similar decrease in LOHS by 2-3 days was reported in patients who underwent emergency surgery and were managed by ERAS protocols, which have been documented [8-10,16]. In the present study, a significant reduction in the length of hospital stay was found in the ERAS group when compared to the standard care group (RR=61.2; p=0.000). The mean length of hospital stay of 7.82±3.59 days was significantly higher (p=0.000) in the standard method as compared to ERAS, which was 4.26±0.73 days. Hence, it can be concluded that the results obtained in our study are comparable to the previous literature.

Limitation

The sample size of the study was relatively small. The surgical expertise in laparoscopic duodenal ulcer was not available at the center; hence, all patients underwent open Graham patch repair. ERAS protocols are composed of the use of minimally invasive surgical techniques. In the present study, we wanted to investigate the safety of the application of these protocols in an open Graham patch repair and confirm its feasibility in the setup. The study might have favorable results due the exclusion of patients with a poor surgical outcome (patients with multiple perforations, perforation size of >1 cm, and ASA categories 3 and 4). The results should be interpreted with caution as in the stipulated study period, and the exclusion criteria resulted in a small sample size. However, this study aimed at evaluating the efficacy of the ERAS pathway in patients undergoing emergency abdominal surgery for perforated duodenal ulcers. The effective application of ERAS protocols in the emergency subgroup of these patients can be concluded. There is a need for a similar large multicentric trial to safely and effectively establish the standard protocols for the successful application of ERAS protocols in an emergency scenario.

## Conclusions

ERAS protocols can be successfully applied to the selected subgroup of patients undergoing emergency open Graham patch repair for perforated duodenal ulcers. The early removal of nasogastric tubes and the early initiation of feeding can significantly decrease the length of hospital stay and reduce the patient burden on the hospital. Thus, it may be considered safe to apply ERAS protocols with certain modifications in such a selected group of patients. However, the application of ERAS pathways in emergency setup needs to be further evaluated in order to develop standardized protocols for a surgical emergency group of patients. This study further reinforces the established literature on the application of ERAS pathways in an emergency setup.
